# Recent advances in the understanding and treatment of pemphigus and pemphigoid

**DOI:** 10.12688/f1000research.14474.1

**Published:** 2018-08-30

**Authors:** Jun Yamagami

**Affiliations:** 1 Department of Dermatology, Keio University School of Medicine, 35 Shinanomachi, Shinjuku-ku, Tokyo, 160-8582, Japan

**Keywords:** pemphigus, pemphigoid

## Abstract

Pemphigus and pemphigoid are characterized as autoimmune blistering diseases in which immunoglobulin G autoantibodies cause blisters and erosions of the skin or mucosa or both. Recently, understanding of the pathophysiology of pemphigus and pemphigoid has been furthered by genetic analyses, characterization of autoantibodies and autoreactive B cells, and elucidation of cell–cell adhesion between keratinocytes. For the management of pemphigus and pemphigoid, the administration of systemic corticosteroids still represents the standard treatment strategy; however, evidence of the efficacy of therapies not involving corticosteroids, such as those employing anti-CD20 antibodies, is increasing. The goal should be to develop antigen-specific immune suppression-based treatments.

## Introduction

Autoimmune blistering diseases are induced by autoantibodies against components of the skin. There are two main types of autoimmune blistering disease: pemphigus (blister in the epidermis) and pemphigoid (subepidermal blistering). In pemphigus, autoantibodies target desmoglein 1 (Dsg 1) and Dsg 3, which play a major role in desmosomes essential for cell–cell adhesion between keratinocytes and cause blister formation with acantholysis ^1^. In pemphigoid, autoantibodies target molecules involved in connecting basal epithelial cells to the basement membrane in hemidesmosomes, such as type XVII collagen (COL17, BP180), dystonin-e (BP230), type VII collagen (COL7), p200, and laminin 332 ^2, 3^. This article reviews recent advances in our understanding of the pathophysiology and treatment of pemphigus and pemphigoid.

## Pemphigus

### Pathophysiology

Pemphigus is considered one of the best-characterized human autoimmune diseases. Recent studies have shed light on the genetic susceptibility to pemphigus, characterized by the presence of Dsg-reactive autoantibodies, and revealed the mechanisms underlying blister formation ^4^.

***Genetic susceptibility.*** Several human leukocyte antigen (HLA) alleles, such as HLA-DRB1*0402 and HLA-DQB1*0503, ** have been suggested as risk factors for pemphigus vulgaris (PV) ^5– 7^. Although recent microarray analyses have indicated that HLA status may be a key driver of autoantibody expression and could be related to disease activity via the oxidative stress that occurs during PV, the correlation between the HLA genetic profile and overall clinical profile remains unclear ^8, 9^. A non-HLA marker encoding ST18, a molecule that regulates apoptosis and inflammation, has been investigated as a new candidate gene associated with PV. *ST18*-associated variants may predispose to PV in a population-specific manner, and an association between *ST18* and PV was found in Jewish and Egyptian, but not German, populations ^10^. In one report, PV serum-induced secretion of key inflammatory molecules by keratinocytes was related to a PV-associated functional risk variant residing within the *ST18* promoter region ^11^.

***Characterization of autoantibodies.*** Investigations of anti-Dsg autoantibodies and autoreactive B cells have helped to uncover the mechanisms underlying the development of pemphigus and the production of autoantibodies ^12– 15^. Recent studies suggested that B cells with *V _H_1-46* heavy chain gene usage might be prone to Dsg3 autoreactivity. Dsg3-specific B cells that use *V _H_1-46* demonstrated relatively few somatic mutations and required few, or none, of these mutations to bind Dsg3 ^16^. Furthermore, in patients with PV, cross-reactivity of B cells with *V _H_1-46* to both Dsg3 and VP6, a rotavirus capsid protein, suggests that the anti-Dsg B-cell repertoire can cross-react with foreign antigens ^17^. Previously, anti-Dsg1 immunoglobulin G (IgG) monoclonal antibodies that can cross-react with LJM11, a sand fly antigen, were reported in the endemic form of pemphigus foliaceus ^18^. These findings indicate that the immune response to foreign antigens may lead to autoimmunity, in turn resulting in the development of pemphigus.

Long-term analysis of autoreactive B-cell repertoires in patients with pemphigus revealed the presence of identical anti-Dsg antibody clones before and after treatment, suggesting that targeting specific sets of autoreactive B cells may be a feasible therapeutic strategy ^19^. However, proteomic analysis of pemphigus autoantibodies gave us a different perspective by indicating that changes in the proportions of autoantibodies occur over time ^20^. Further research is necessary to fully delineate the mechanisms of autoantibody production in pemphigus. In addition to Dsg, other autoimmune targets have been found, and their pathogenic roles might be elucidated in future investigation ^21^.

***Blister formation by autoantibodies.*** A direct pathogenetic role of autoantibodies in pemphigus has been clearly established. There are two major routes to acantholytic blister formation: autoantibody-mediated steric hindrance of desmosomal adhesion ^12, 15, 22^ and activation of particular cell signaling pathways such as the p38 mitogen-activated protein kinase (MAPK) pathway ^23– 26^. Studies have shown that pathogenic anti-Dsg monoclonal antibodies can bind directly to residues that mediate adhesion and that polyclonal antibodies contribute to acantholysis in a non-redundant way; thus, all antibodies have the potential to cause blistering via synergistic mechanisms ^27, 28^. Advances in understanding of the crystal structure of desmocollins and Dsgs ^29^ and in techniques for high-resolution imaging of the skin will further clarify the pathophysiological regulation of keratinocyte cell–cell adhesion in pemphigus ^26, 30^.

### Treatment

The goal in pemphigus treatment is to maintain complete remission, defined as the absence of new or established lesions ^31^. Ideally, all systemic therapy should be stopped; however, remission achieved by minimal therapy, prednisone (≤10 mg/day), or minimal adjuvant therapy (or a combination of these) may be a more realistic goal in the management of pemphigus ^32, 33^.

Because of the rarity of pemphigus, published guidelines for its management rely mostly on expert consensus, except for a few evidence-based controlled studies. Treatments recommended by European and Japanese guidelines include corticosteroids and immunosuppressive reagents, such as azathioprine and cyclophosphamide, plasmapheresis, intravenous immunoglobulin (IVIG), and rituximab; most therapies aim to improve symptoms by reducing serum autoantibodies, either directly or through generalized immune suppression ^33, 34^. Recent studies have highlighted the advantages of rituximab, a monoclonal anti-CD20 antibody that targets CD20 ^+^ B cells, in the treatment of pemphigus ^35– 38^. The results of multicenter prospective randomized trials of rituximab, as a first-line treatment for moderate to severe cases of pemphigus, were recently published in France ^39^. At 2 years after treatment, 41 (89%) of 46 patients assigned to rituximab plus short-term prednisone were in complete remission and off therapy versus 15 (34%) of 44 patients assigned to the prednisone-alone treatment. Significantly more serious adverse events (that is, of grade 3–4) were reported in the prednisone-alone group than in the rituximab-plus-prednisone group. These results suggest that first-line use of rituximab for patients with pemphigus is more effective, and causes fewer adverse events, than corticosteroids alone. As such, the treatment strategy for pemphigus may change worldwide.

The ideal therapy for pemphigus would eliminate only those autoantibodies produced by Dsg-specific B cells. A recent study suggested the feasibility of such targeted therapy through the use of chimeric antigen receptor T (CART) cells ^40^. In PV, autoantigen-based chimeric immunoreceptors can direct T cells to kill autoreactive B cells according to the specificity of the B-cell receptor (BCR). Human T cells engineered to express chimeric autoantibody receptor (CAAR), consisting of Dsg3 fused to CD137-CD3ζ signaling domains (Dsg3 CAAR-T cells), exhibit specific cytotoxicity against cells expressing anti-Dsg3 BCRs *in vitro* and *in vivo*.

## Pemphigoid

### Pathophysiology

There are three major types of pemphigoid: bullous pemphigoid (BP), which is the most common autoimmune bullous disorder and is characterized by widespread erythema and blisters on the skin; mucous membrane pemphigoid, which affects mainly the oral mucosa; and epidermolysis bullosa acquisita, which is a bullous disease associated with autoimmunity to COL7, the major component of the anchoring fibrils of the dermal–epidermal junction.

***Disease development.*** Although the genetic background and mechanism underlying the development of pemphigoid remain to be precisely determined, several studies have suggested a genetic predisposition in its etiology ^41– 43^. A German study showed that a polymorphism in the mitochondrially encoded ATP synthase 8 gene ( *MT-ATP8*) may have an association with BP pathogenesis, which suggests a contribution of mitochondrial abnormalities to BP immunopathology ^44^. Furthermore, a recent study showed that gene polymorphism in *CYP2D6*, which is among the cytochrome P450 isoenzymes, may influence the risk of drug-induced BP ^45^. Dipeptidyl peptidase-IV (DPP-4) inhibitors have been increasingly implicated as a cause of drug-induced BP. Recently, a strong association between the use of DPP-4 inhibitors and the risk of BP was reported ^46^. Although the mechanism is still unclear, several reports revealed that patients with BP associated with the use of DPP-4 inhibitors tended to exhibit a non-inflammatory clinical phenotype and possessed IgG autoantibodies against full-length COL17, albeit outside of the non-collagenous 16A domain (NC16A) (the major IgG epitope in typical BP) ^47, 48^.

***Blister formation by autoantibodies.*** Following binding of IgG autoantibodies to their target antigens in the basement membrane zone (BMZ), complements and other factors induce neutrophil and eosinophil chemotaxis and mast cell degranulation as well as release their proteolytic enzymes, such as metallopeptidase-9 and neutrophil elastase ^49– 51^. Although these complement-dependent mechanisms are believed to be essential for the inflammatory cascade that causes subepidermal blistering, evidence supporting blister induction via direct interaction between autoantibodies and autoantigens (complement-independent mechanism) has recently been reported ^52– 57^.

IgG4 is assumed to lack the ability to fix complement, and BP cases in which IgG4 is dominant and C3 deposition at the BMZ is lacking have been reported ^58^. Additionally, passive transfer of IgG4 and IgG1 autoantibodies in patients with BP has been shown to induce blister formation in neonatal C3-deficient COL17-humanized mice without complement activation ^59^. These reports suggest that IgG4 autoantibodies may contribute to complement-independent blister formation in some cases of BP.

Recent studies also revealed that skin fragility in BP is induced by macropinocytosis of immune complexes after the binding of autoantibodies to COL17 on the cell surface ^60^. Studies showing that antibodies targeting areas outside of NC16A had less capacity to deplete COL17 on keratinocytes than antibodies targeting NC16A suggest that the pathogenicity of autoantibodies in BP could be epitope dependent ^61, 62^. Recent studies have offered some biomarkers for more efficient diagnosis and prediction of prognosis in pemphigoid ^63, 64^.

### Treatment

Systemic glucocorticoids remain the standard treatment for pemphigoid. Superpotent topical corticosteroids have shown efficacy in BP but may not be practical for daily use as whole-body ointments ^65, 66^. British guidelines have mentioned anti-inflammatory antibiotics (that is, tetracyclines) for treating BP, but no clear evidence supporting this recommendation was provided ^67^. Recently, a randomized controlled trial comparing doxycycline with prednisolone as an initial treatment for BP was published ^68^. Participants were randomly assigned to a doxycycline (200 mg/day) or a prednisolone (0.5 mg/kg per day) group, and 83 (74%) of 112 patients had three or fewer blisters at 6 weeks compared with 92 (91%) of 101 patients on prednisolone. The rate of severe, life-threatening, or fatal events at 52 weeks were 18% (22 of 121) for those on doxycycline and 36% (41 of 113) for those on prednisolone. This study provided important evidence that doxycycline is not inferior to oral prednisolone for short-term blister control in BP and also is significantly safer in the long term. Although disease severity should be carefully evaluated on a case-by-case basis and the mechanism of action of doxycycline is still unclear, this agent likely will be widely used as a first-line treatment for BP.

Most therapies that have shown efficacy against pemphigus have also been tried for pemphigoid. A randomized double-blind trial of IVIG showed that the disease activity score on day 15 was significantly lower in severe BP versus the baseline value, suggesting that IVIG could be a good option for patients resistant to corticosteroids ^69, 70^. In a retrospective study, first-line combination therapy with rituximab and corticosteroids was recommended for cases of moderate to severe BP; the regimen achieved a higher rate of complete remission compared with treatment by rituximab ^71, 72^.

Accumulating evidence suggests a pathogenic role for IgE in BP. IgE deposition in the BMZ in patients with BP has frequently been observed ^73, 74^, and a correlation between serum levels of IgE autoantibodies against COL17 and BP disease activity has been reported ^75^. High-affinity IgE receptor (FcεRI)-bound IgE on mast cells and eosinophils may be involved in the degranulation of such cells following their capture of shed extracellular fragments of COL17, in turn resulting in the development of urticarial erythema in BP ^76^. Reports of BP cases successfully treated with anti-IgE monoclonal antibodies also support the potential of IgE as a therapeutic target in BP ^77, 78^.

## Closing remarks

The precise mechanism that triggers autoantibody production in patients with pemphigus and pemphigoid remains unknown. Elucidating the pathogenesis of these diseases via repertoire analysis and study of the mechanism of activation of autoreactive B cells is a promising approach. Furthermore, clarification of the mechanism underlying blister formation may yield a novel treatment target. Therapeutic approaches that do not involve corticosteroids, such as those based on anti-CD20 antibodies, are gradually being established. A goal of treatments for pemphigus and pemphigoid should be targeted depletion only of antigen-specific T/B cells or autoantibodies.
